# A Challenging Case Report of Severe Asherman’s Syndrome: Managing Uterine Perforation During Surgery

**DOI:** 10.1155/crog/8897371

**Published:** 2026-04-16

**Authors:** Khishotul Hayati, Artha Falentin Putri Susilo, Mulyanusa Amarullah Ritonga, Tono Djuwantono

**Affiliations:** ^1^ Department of Obstetrics and Gynecology, Faculty of Medicine, Padjadjaran University, Bandung, Indonesia, unpad.ac.id

**Keywords:** Asherman’s syndrome, case report, hysteroscopic adhesiolysis, uterine perforation

## Abstract

**Introduction:**

Asherman’s syndrome (AS), characterized by intrauterine adhesions (IUAs), can significantly distort uterine anatomy and complicate hysteroscopic adhesiolysis. Although hysteroscopic adhesiolysis is the standard treatment, severe adhesions increase the risk of complications such as uterine perforation.

**Case Illustration:**

We report a 31‐year‐old woman with severe AS who developed uterine perforation during hysteroscopic adhesiolysis. During dissection of dense adhesions, a small fundal perforation (0.5 cm × 0.5 cm) was suspected after sudden loss of resistance and bleeding. The procedure was immediately halted, and diagnostic laparoscopy confirmed a full‐thickness fundal defect that was repaired laparoscopically. After stabilization, hysteroscopic management was resumed with placement of an amnion graft to promote endometrial regeneration. However, intraoperative bleeding recurred, and reassessment revealed enlargement of the perforation. Repeat laparoscopy confirmed a larger fundal defect, which was repaired with definitive laparoscopic hysterorrhaphy. The patient recovered uneventfully, received postoperative hormonal therapy, and resumed menstruation 1 month after surgery.

**Conclusion:**

This case highlights the risk of uterine perforation during adhesiolysis in severe AS and emphasizes the importance of early recognition and prompt laparoscopic management. Adjunctive guidance techniques and combined hysteroscopic–laparoscopic approaches may improve procedural safety and help preserve uterine integrity.

## 1. Introduction

Asherman’s syndrome (AS) is a condition characterized by the presence of intrauterine adhesions (IUAs), which form within the uterine cavity and/or endocervix. These adhesions can lead to a range of clinical manifestations, including amenorrhea, hypomenorrhea, recurrent pregnancy loss, infertility, and abnormal placentation. The syndrome often results from trauma to the endometrium, commonly following uterine surgical procedures [1].

The syndrome is particularly associated with dilation and curettage (D&C) procedures. It occurs in approximately 13% of women undergoing a first‐trimester termination of pregnancy and up to 30% of women undergoing D&C following a late spontaneous abortion [1]. This enhanced diagnostic capability emphasizes the need for greater awareness and early identification of AS, particularly in patients with a history of uterine trauma or symptoms suggestive of IUAs [2].

Hysteroscopic adhesiolysis is the preferred treatment to restore uterine cavity integrity. However, this procedure carries risks, where dense fibrotic bands, cavity obliteration, and loss of normal anatomical landmarks significantly increase procedural complexity and operative risk, notably iatrogenic uterine perforation, which can complicate treatment. The incidence of uterine perforation during hysteroscopic adhesiolysis varies, with some studies reporting rates as low as 0.9%. Despite its rarity, the potential for significant complications necessitates a comprehensive understanding of both AS and the management of associated risks during surgical intervention [3]. We present a challenging case of severe AS complicated by a large fundal uterine perforation during hysteroscopic adhesiolysis, successfully managed with immediate laparoscopic repair. This report aims to highlight current risk factors, intraoperative warning signs, and surgical principles for the prevention and management of uterine perforation, reinforcing the role of combined hysteroscopic and laparoscopic approaches in complex cases.

## 2. Case Presentation

A 31‐year‐old woman (P1A0, with one living child aged 9 years) presented with a chief complaint of amenorrhea persisting for the past 9 years. Her obstetric history includes one full‐term delivery in 2015, followed by two curettage procedures performed 2 weeks apart due to retained placental tissue. She denied experiencing abdominal pain, dysmenorrhea, postcoital bleeding, abdominal masses, or any notable changes in weight or appetite.

On physical examination, vital signs were within normal limits and her body mass index was within the normal range. Abdominal palpation revealed no tenderness, guarding, rebound tenderness, or palpable masses. Pelvic examination showed a normal vulva and vagina, with a smooth and firm cervix and an external os that admitted one finger.

**Figure 1:**
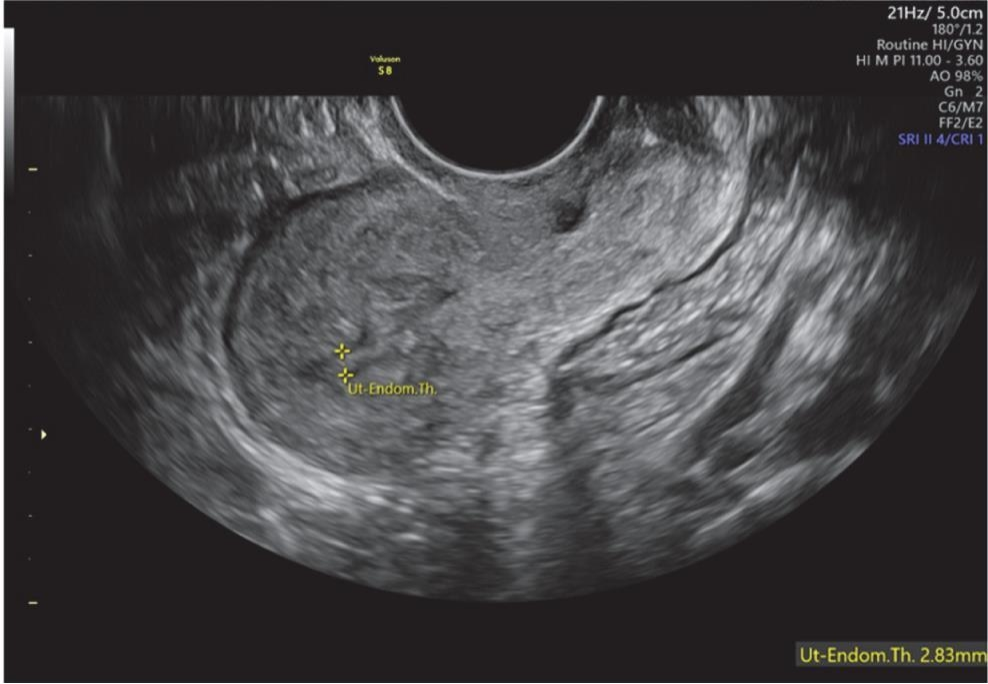
Transvaginal two‐dimensional gray scale ultrasonography of the uterus in the sagittal (longitudinal) plane.

Transvaginal two‐dimensional gray scale ultrasonography (Figure 1) of the uterus in the sagittal (longitudinal) plane. The image demonstrates a markedly thin and irregular endometrial lining, with an endometrial thickness of approximately 2.83 mm (yellow calipers), consistent with severe AS.

The uterine corpus, fundus, and cervical canal are visualized as key anatomical landmarks. The indistinct endometrial–myometrial interface and reduced endometrial cavity volume support the diagnosis of severe AS. Laboratory tests conducted showed the following results: AMH 8.48 ng/mL, estradiol 65.41 pg/mL, FSH 6.84 mIU/mL, LH 8.74 mIU/mL, prolactin 9.27 ng/mL, and progesterone 0.90 ng/mL.

The management plan included a scheduled operative hysteroscopy combined with an amnion graft procedure.

## 3. Operative Procedure

**Figure 2:**
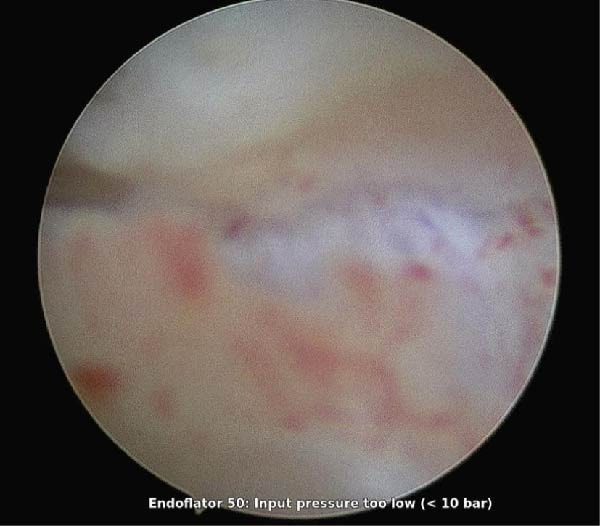
Hysteroscopic view of cervical stenosis.

The procedure was performed under general anesthesia with the patient in the lithotomy position. Following sterile preparation, the cervix was grasped with a tenaculum. Diagnostic hysteroscopy was initiated using a vaginoscopic approach; however, the hysteroscope could only be advanced up to the external cervical os due to cervical stenosis (Figure 2).

Intraoperative hysteroscopic image obtained using a rigid hysteroscope during operative hysteroscopy. The circular field represents the endoscopic view through the cervical canal, demonstrating marked cervical stenosis with dense fibrotic tissue, resulting in a narrowed lumen and poor visualization of the uterine cavity. The whitish fibrous bands and irregular mucosal surface indicate chronic scarring, consistent with severe IUAs. Limited distention is evident, reflecting resistance to distension medium flow during cervical entry.

**Figure 3:**
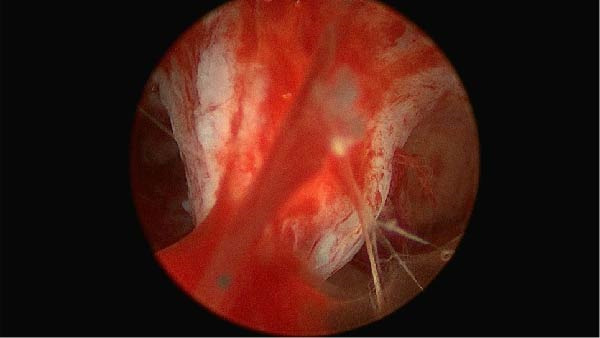
Hysteroscopic view of dense intrauterine adhesions involving the anterior uterine cavity.

Initial vaginoscopic entry was limited by significant cervical stenosis; progressive cervical dilation was therefore performed using Hegar dilators from size 5–7 followed by uterine sounding, which revealed a retroflexed uterus measuring 7 cm in depth. Dilation was performed gently with tactile feedback; no forced dilatation was attempted. A Covidien TruClear Elite hysteroscope with an integrated working channel (Medtronic, Minneapolis, MN, USA; outer diameter 5 mm, 30° lens) was introduced under direct visualization. Normal saline (0.9% isotonic) was used as distention medium and delivered via an automated pressure‐controlled pump (continuous flow); intrauterine pressure was maintained at approximately 80–100 mmHg with a flow rate of 200–300 mL/min. Initial visualization revealed near‐complete obliteration of the uterine cavity by dense, fibrotic adhesions with loss of normal anatomical landmarks. Cavity distention was suboptimal, and visualization was limited. IUAs were visualized, confirming the diagnosis of AS (Figure 3).

Intrauterine evaluation showing dense fibrotic scar tissue located in the anterior third of the uterine cavity, extending from the anterior uterine wall toward the posterior wall, resulting in partial obliteration of the uterine cavity. The whitish, avascular fibrous bands with surrounding hyperemic endometrium are characteristic of severe IUAs, with distortion of normal uterine cavity anatomy and limited visualization of intrauterine landmarks. Based on the hysteroscopic findings (extensive dense adhesions, involvement of the uterine cavity > 3/4, and significant obliteration of the endometrial surface), this case is now clearly described as severe AS according to the American Fertility Society (AFS) classification.

**Figure 4:**
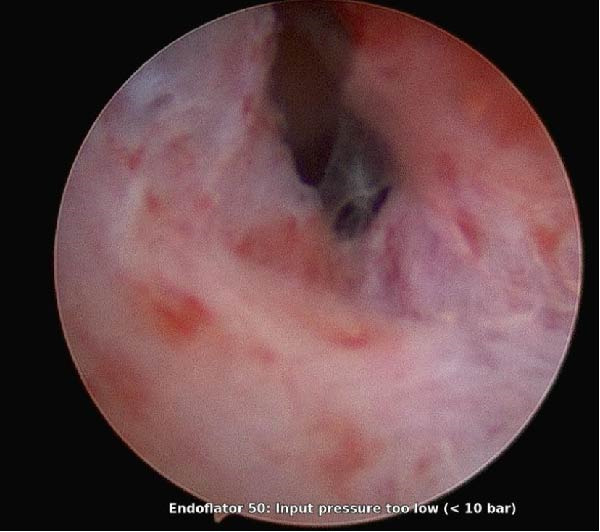
Hysteroscopic image showing an irregular defect at the uterine fundus with disrupted endometrium, consistent with uterine perforation during operative hysteroscopy.

Adhesiolysis was performed through the TruClear working channel using cold scissors. Dissection began at the lower uterine segment and progressed cranially along the presumed midline with low‐pressure distention and minimal traction. Tissue planes were assessed continuously; during division of dense fundal adhesions the operator encountered a sudden loss of resistance with an abrupt increase in apparent cavity depth and deterioration of hysteroscopic visualization, consistent with suspected full‐thickness perforation. No active bleeding was observed hysteroscopically (Figure 4).

## 4. First Laparoscopic Assessment and Repair

**Figure 5:**
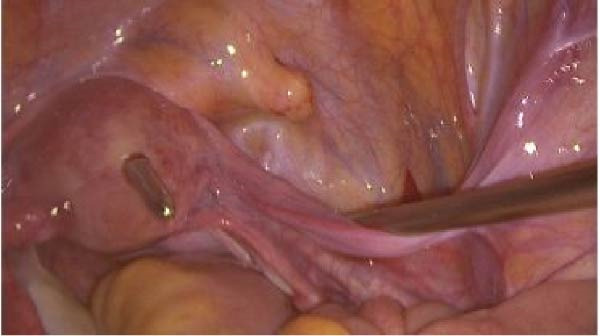
Laparoscopic image demonstrating a full‐thickness perforation at the uterine fundus identified during hysteroscopic adhesiolysis.

At the moment of suspected perforation the patient remained hemodynamically stable. The hysteroscopic procedure was immediately discontinued, and diagnostic laparoscopy was performed. Laparoscopic evaluation confirmed a 0.5 cm × 0.5 cm full‐thickness perforation at the uterine fundus with minimal hemoperitoneum and no associated visceral injury (Figure 5). The defect was repaired laparoscopically using interrupted 2–0 polyglactin (Vicryl) sutures. Port configuration included a 10 mm umbilical camera port and two 5 mm working ports in the lower quadrants. Hemostasis was confirmed, the peritoneal cavity irrigated and suctioned, and uterine integrity verified laparoscopically. After confirming uterine integrity, the procedure was concluded.

## 5. Amnion Graft Insertion

**Figure 6:**
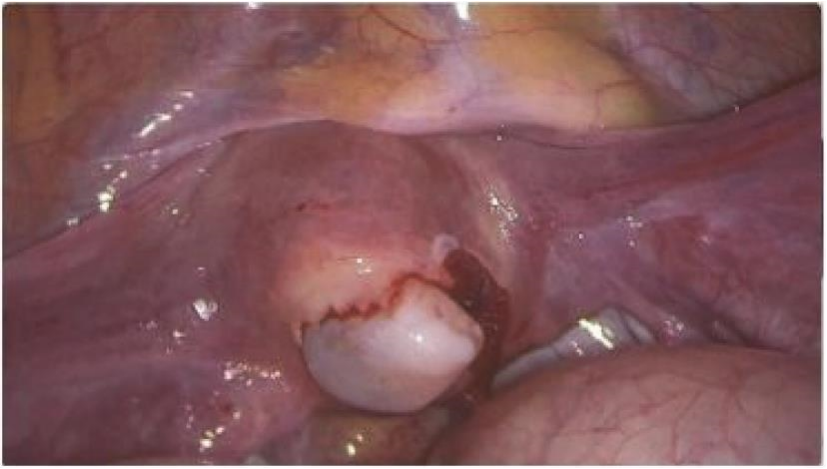
Laparoscopic image showing extension of a previously repaired fundal uterine perforation identified during intraoperative reassessment.

After stabilization and laparoscopic reassessment, an amnion graft was attempted for endometrial regeneration and adhesion prevention. The graft was temporarily secured with a size‐10 silicone Foley catheter and gentle balloon inflation (balloon volume 5 mL). During balloon inflation and graft manipulation, brisk intrauterine bleeding occurred and hysteroscopic reassessment showed enlargement of the fundal defect to approximately 3 cm × 3 cm. We believe mechanical stress from balloon inflation/manipulation on a fresh fundal repair likely contributed to suture pull‐through and extension of the defect, prompting immediate repeat laparoscopy (Figure 6).

## 6. Second Laparoscopic Repair

**Figure 7:**
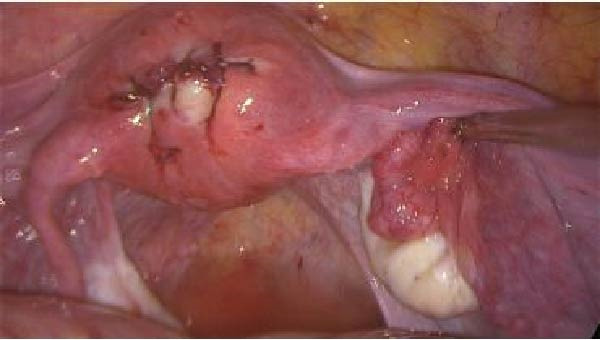
Laparoscopic image showing fundal uterine repair using size‐1 V‐Loc sutures in a figure‐of‐eight configuration.

The procedure was once more halted, and immediate laparoscopy was performed. Laparoscopic findings confirmed a larger fundal uterine perforation measuring approximately 3 cm × 3 cm, corresponding to the site of the previous repair. Definitive laparoscopic hysterorrhaphy of the expanded 3 cm × 3 cm defect was performed using size‐1 V‐Loc barbed sutures (Medtronic) in a figure‐of‐eight configuration with multiple full‐thickness bites to ensure secure myometrial approximation (Figure 7). A tension‐free closure was achieved and hemostasis confirmed; no adjacent organ injury was identified. This resulted in successful laparoscopic hysterorrhaphy without the need for hysterectomy.

Postoperatively, the patient received intravenous ceftriaxone 2 gr daily and paracetamol 500 mg tid. To facilitate endometrial regeneration, prevent readhesion, and avoid pregnancy during the uterine healing period following repeated fundal repair, hormonal therapy was initiated. The patient was prescribed sequential estrogen–progestin therapy, consisting of oral estradiol valerate 2 mg twice daily for 21 days, followed by medroxyprogesterone acetate 10 mg daily for the last 10 days of the cycle, for a total duration of 3 months. This regimen was selected to promote endometrial proliferation while ensuring regular withdrawal bleeding and protecting against unopposed estrogen exposure. The patient was counseled to avoid conception during this period to allow adequate myometrial healing after laparoscopic hysterorrhaphy. The postoperative course was uneventful, with no signs of infection or recurrent bleeding. At 1 month following adhesiolysis, the patient had resumed menstruation. A second‐look hysteroscopy was scheduled after completion of postoperative hormonal therapy; however, this evaluation was not carried out because the patient was lost to follow‐up and did not return for the planned 3‐month visit.

## 7. Discussion

Severe IUAs in advanced Asherman syndrome frequently distort the uterine cavity and obscure normal anatomical landmarks, making hysteroscopic adhesiolysis technically challenging [4]. In such situations, the cavity may become markedly narrowed or essentially obliterated, limiting distention and impairing visualization of the endometrial surface and tubal ostia; these conditions increase the risk of misidentifying the correct dissection plane and consequently raise the likelihood of uterine perforation. Previous series and reviews report higher perforation risk during hysteroscopic procedures when dense IUAs severely distort the cavity [3, 5].

In the present case, the initial fundal perforation was most likely related to near‐complete cavity obliteration with dense fibrotic adhesions at the fundus. Under these circumstances, what appeared hysteroscopically as a central adhesion band may, in fact, represent thin residual myometrium closely adherent to serosa. During cold‐scissor adhesiolysis, advancement of the instrument through this attenuated tissue can produce a sudden loss of resistance and unintended full‐thickness perforation—a mechanism described in prior reports of difficult adhesiolysis [3, 6]. In this patient, the presumed midline dissection proved unreliable because anatomical landmarks (including the tubal ostia) were not visible; consequently, accurate orientation was not possible and scissors likely entered attenuated fundal myometrium, producing the observed breach.

After laparoscopic repair of the initial perforation, the subsequent enlargement of the fundal defect likely reflected limited residual myometrial thickness combined with mechanical stress during attempted intrauterine manipulation [7]. Amnion graft placement secured with a Foley catheter and balloon inflation was intended to promote endometrial regeneration and prevent readhesion, but balloon expansion immediately after repair likely contributed to suture pull‐through and extension of the defect. Although intrauterine balloon stenting is commonly used after adhesiolysis to maintain cavity patency, its application across a fresh fundal repair can place axial and shear forces on the sutured tissue and should be used with extreme caution (if used at all, minimal volumes of 3–5 mL are advised) [8, 9].

Management decisions depend on perforation size and patient stability. Small, uncomplicated perforations may be observed, whereas larger full‐thickness defects generally require surgical repair. Laparoscopic hysterorrhaphy offers direct visualization, organ assessment and secure myometrial closure while preserving the uterus; in this case prompt laparoscopic repair achieved hemostasis and uterine preservation without hysterectomy [7].

This case suggests three practical recommendations for similar patients: (1) anticipate severe cavity distortion in advanced Asherman’s and arrange real‐time ultrasound support or plan for early combined hysteroscopic–laparoscopic access; (2) if a perforation is repaired, avoid immediate forceful intrauterine balloon inflation or aggressive graft manipulation across the suture line and confirm repair integrity laparoscopically before further intrauterine work; (3) when feasible, use layered, deep myometrial bites for definitive closure to reduce the risk of suture pull‐through [4, 5, 7].

This case has several limitations. Intraoperative ultrasound guidance was not utilized due to the unavailability of the required equipment and personnel in the operating theater. In addition, second‐look hysteroscopy was not performed because the patient did not return for the scheduled 3‐month follow‐up, limiting objective evaluation of postoperative uterine cavity restoration and the presence of residual or recurrent adhesions. This experience underscores the importance of considering adjunctive imaging or a staged surgical approach when managing severe AS with obliterated intrauterine landmarks, as these strategies may improve spatial orientation during adhesiolysis and reduce the risk of fundal perforation while preserving uterine integrity.

## 8. Conclusion

Severe AS presents significant surgical challenges due to distorted uterine anatomy and loss of normal intrauterine landmarks. Although hysteroscopic adhesiolysis remains the standard treatment, the risk of complications such as uterine perforation increases in cases with dense and extensive adhesions. This case highlights the importance of early recognition and prompt surgical management of perforation to prevent further morbidity and preserve uterine integrity. The use of adjunctive guidance techniques, including ultrasound or combined hysteroscopic–laparoscopic approaches, may improve spatial orientation and procedural safety in complex cases. With careful surgical technique and appropriate postoperative strategies to prevent adhesion recurrence, restoration of uterine anatomy and preservation of reproductive potential remain achievable.

## Author Contributions

Khishotul Hayati wrote the proposal, gave training on data collection, analyzed the data, and drafted the paper. Artha Falentin Putri Susilo, Mulyanusa Amarullah Ritonga, and Tono Djuwantono approved the proposal with some revisions and participated in data analysis and manuscript writing.

## Funding

The authors received no financial support for the research, authorship, and/or publication of this article.

## Disclosure

All authors have read and approved the final manuscript.

## Ethics Statement

The authors’ institution does not require ethical approval for publication of single case report.

## Consent

The patient provided written informed consent for publication of the case report and the accompanying images.

## Conflicts of Interest

The authors declare no conflicts of interest.

## Data Availability

Data, models, and code supporting this study’s findings are available from the corresponding author.
